# Crystal structure and the DFT and MEP study of 4-benzyl-2-[2-(4-fluoro­phen­yl)-2-oxoeth­yl]-6-phenyl­pyridazin-3(2*H*)-one

**DOI:** 10.1107/S2056989019008557

**Published:** 2019-06-21

**Authors:** Said Daoui, Md. Serajul Haque Faizi, Fouad El Kalai, Rafik Saddik, Necmi Dege, Khalid Karrouchi, Noureddine Benchat

**Affiliations:** a Laboratory of Applied Chemistry and Environment (LCAE), Department of Chemistry, Faculty of Sciences, University Mohamed Premier, Oujda 60000, Morocco; b Department of Chemistry, Langat Singh College, Babasaheb Bhimrao Ambedkar Bihar University, Muzaffarpur, Bihar-842001, India; c Laboratory of Organic Synthesis, Extraction and Development, Faculty of Sciences, Hassan II University, Casablanca, Morocco; d Ondokuz Mayıs University, Faculty of Arts and Sciences, Department of Physics, 55139, Kurupelit, Samsun, Turkey; e Laboratory of Plant Chemistry, Organic and Bioorganic Synthesis, URAC23, Faculty of Science, BP 1014, GEOPAC Research Center, Mohammed V University, Rabat, Morocco

**Keywords:** crystal structure, pyridazin-3(2*H*)-one, hydrogen bonding, C–H⋯π inter­action

## Abstract

The title pyridazin-3(2*H*)-one derivative, C_25_H_19_FN_2_O_2_, crystallizes with two independent mol­ecules (*A* and *B*) in the asymmetric unit. In mol­ecule *A*, the 4-fluoro­phenyl ring, the benzyl ring and the phenyl ring are inclined to the central pyridazine ring by 86.54 (11), 3.70 (9) and 84.857 (13)°, respectively. In mol­ecule *B*, the corresponding dihedral angles are 86.80 (9), 10.47 (8) and 82.01 (10)°, respectively. In the crystal, the *A* mol­ecules are linked by pairs of C—H⋯F hydrogen bonds, forming inversion dimers with an *R*_2_^2^(28) ring motif. The dimers are linked by C—H⋯O hydrogen bonds and a C—H⋯π inter­action, forming columns stacking along the *a*-axis direction. The *B* mol­ecules are linked to each other in a similar manner and form columns separating the columns of *A* mol­ecules.

## Abstract 2

The title pyridazin-3(2*H*)-one derivative, crystallizes with two independent mol­ecules in the asymmetric unit. The two mol­ecules differ essentially in the orientation of the benzyl ring with respect to the central pyridazine ring; this dihedral angle being 3.70 (9) ° in one mol­ecule and 10.47 (8) ° in the other.

## Chemical context   

Pyridazin-3(2*H*)-ones are pyridazine derivatives, being constructed about a six-membered ring that contains two adjacent nitro­gen atoms, at positions one and two, and with a carbonyl group at position three. The inter­est in these nitro­gen-rich heterocyclic derivatives arises from the fact that they exhibit a number of promising pharmacological and biological activities. These include anti-oxidant (Khokra *et al.*, 2016 ▸), anti-bacterial and anti-fungal (Abiha *et al.* 2018 ▸), anti-cancer (Kamble *et al.* 2017 ▸), analgesic and anti-inflammatory (Ibrahim *et al.* 2017 ▸), anti-depressant (Boukharsa *et al.* 2016 ▸) and anti-ulcer activities (Yamada *et al.*, 1981 ▸). In addition, a number of pyridazinone derivatives have been reported to have potential as agrochemicals, for example as insecticides (Nauen & Bretschneider, 2002 ▸), acaricides (Igarashi & Sakamoto, 1994 ▸) and herbicides (Aza­ari *et al.*, 2016 ▸). The present work is a part of an ongoing structural study of heterocyclic compounds and their utilization as mol­ecular (Faizi *et al.*, 2016 ▸) and fluorescence (Mukherjee *et al.*, 2018 ▸; Kumar *et al.*, 2017 ▸; 2018 ▸) sensors. Given the inter­est in this class of compounds and the paucity of structural data, the crystal structure analysis of the title pyridazin-3(2*H*)-one derivative has been undertaken, along with a DFT study, in order to gain further insight into the mol­ecular structure.
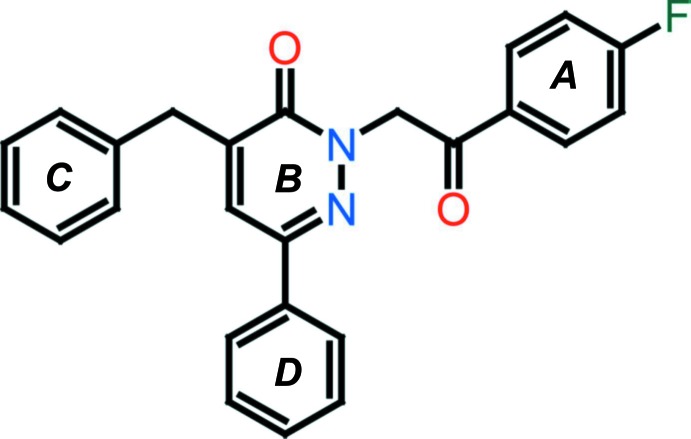


## Structural commentary   

**Figure 1:**
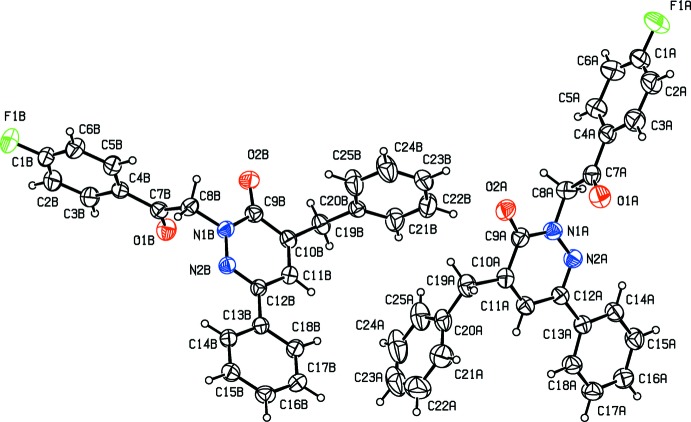
The mol­ecular structure of the title compound, with the atom labelling and displacement ellipsoids drawn at the 30% probability level.

**Figure 2:**
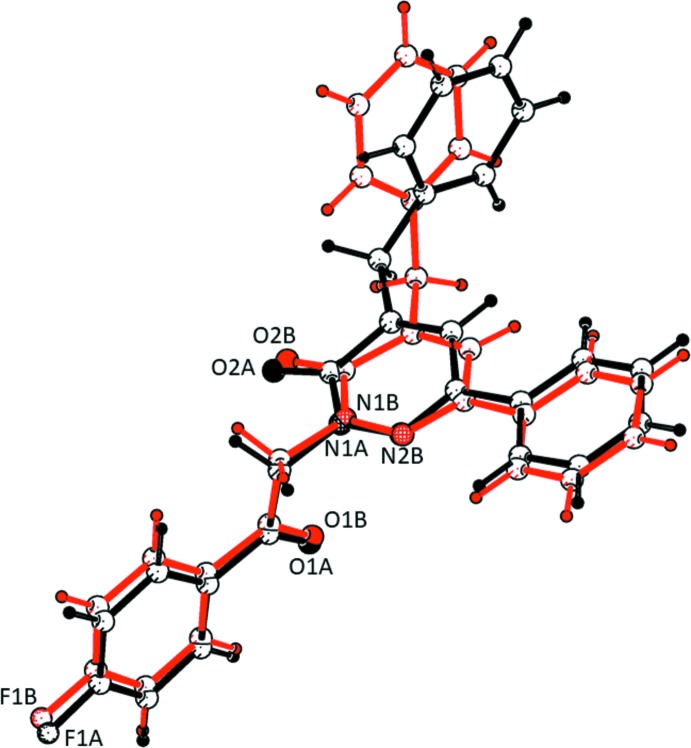
A structural overlap view of mol­ecule *A* (black) on mol­ecule *B* (red), drawn using *PLATON* (Spek, 2009 ▸).

The title compound crystallizes with two independent mol­ecules (*A* and *B*) in the asymmetric unit (Fig. 1 ▸). In each mol­ecule, a central oxopyridazinyl ring is connected to a fluoro­benzyl­acetate group, a phenyl group, and a benzyl residue. The oxopyridazinyl ring (*B*) is planar in both mol­ecules; r.m.s. deviations are 0.029 Å for mol­ecule *A* and 0.009 Å for mol­ecule *B*. In mol­ecule *A*, the 4-fluoro­phenyl ring (*A*; C1*A*–C6*A*), the benzyl ring (*C*; C20*A*–C25*A*) and the phenyl ring (*D*; C13*A*–C18*A*) are inclined to the central pyridazine ring (*B*; N1*A*/N2*A*/C9*A*–C12*A*) by 86.54 (11), 3.70 (9) and 84.87 (13)°, respectively. In mol­ecule *B*, the corresponding dihedral angles are 86.80 (9), 10.47 (8) and 82.01 (10)°, respectively. Hence, the conformation of the two mol­ecules differs essentially in the orientation of the benzyl ring (*C*) with respect to the central pyridazine ring (*B*); 3.70 (9)° in mol­ecule *A* compared to 10.47 (8)° in mol­ecule *B*. The two mol­ecules have an r.m.s. deviation of 0.683 Å for the 30 non-hydrogen atoms (Fig. 2 ▸; *PLATON*; Spek, 2009 ▸).

## Supra­molecular features   

**Figure 3:**
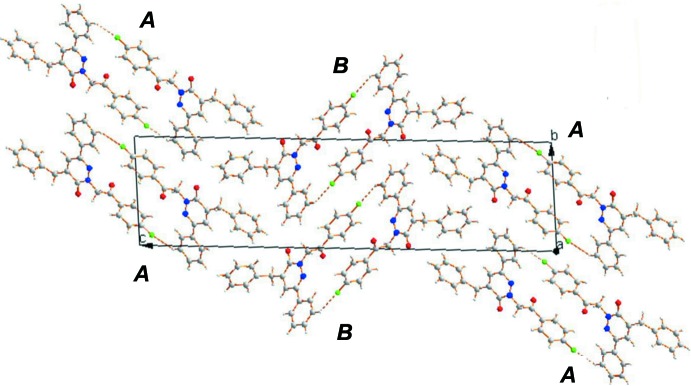
A view along the *a* axis of the crystal packing of the title compound. The C—H⋯F hydrogen bonds are shown as dashed lines (see Table 1 ▸).

In the crystal, the *A* mol­ecules are linked by pairs of C—H⋯F hydrogen bonds, forming inversion dimers with an 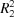
(28) ring motif (Table 1 ▸ and Fig. 3 ▸). The dimers are linked by C—H⋯O hydrogen bonds and a C—H⋯π inter­action (Table 1 ▸), forming columns stacking along the *a*-axis direction. The *B* mol­ecules are linked to each other in a similar manner (Table 1 ▸), and also form columns separating the columns of *A* mol­ecules, as illustrated in Fig. 3 ▸.

## Frontier mol­ecular orbitals analysis   

**Figure 4:**
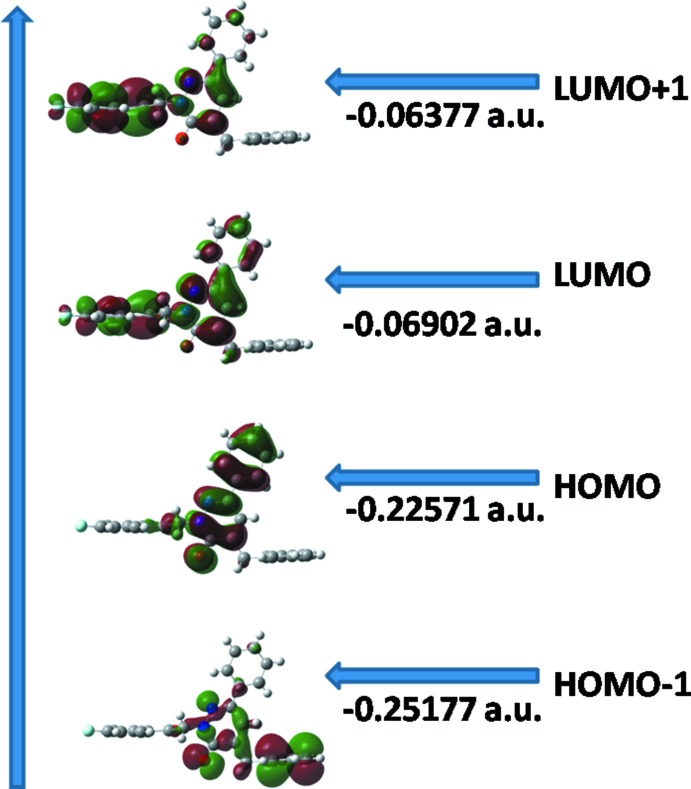
Electron distribution of the HOMO−1, HOMO, LUMO and the LUMO+1 energy levels for the title compound.

The highest occupied mol­ecular orbitals (HOMOs) and the lowest-lying unoccupied mol­ecular orbitals (LUMOs) are named as frontier mol­ecular orbitals (FMOs). The FMOs play an important role in the optical and electric properties, as well as in quantum chemistry and UV–vis spectra. As a result of the inter­action between the HOMO and LUMO orbitals of a structure, a transition state of the π–π* type is observed according to mol­ecular orbital theory. The frontier orbital gap helps characterize the chemical reactivity and the kinetic stability of the mol­ecule. A mol­ecule with a small frontier orbital gap is generally associated with a high chemical reactivity, low kinetic stability and is also termed as a soft mol­ecule. The DFT quantum-chemical calculations for the title compound were performed at the B3LYP/6–311 G(d,p) level (Becke, 1993 ▸) as implemented in *GAUSSIAN09* (Frisch *et al.*, 2009 ▸). The DFT structure optimization was performed starting from the X-ray geometry and the experimental values of the bond lengths and bond angles match the theoretical values. The DFT study shows that the HOMO and LUMO are localized in the plane extending from the whole substituted oxopyridazinyl ring. The electron distribution of the HOMO−1, HOMO, LUMO and LUMO+1 energy levels is shown in Fig. 4 ▸. The HOMO mol­ecular orbital exhibits both *σ* and *π* character, whereas HOMO−1 is domin­ated by *π*-orbital density. The LUMO is mainly composed of *π*-density while LUMO+1 has both *σ* and *π* electronic density. The HOMO–LUMO gap is 0.15669 a.u. and the frontier mol­ecular orbital energies, *E*_HOMO_ and *E*_LUMO_ are −0.22571 and −0.06902 a.u., respectively.

## Mol­ecular electrostatic potential surface analysis   

**Figure 5:**
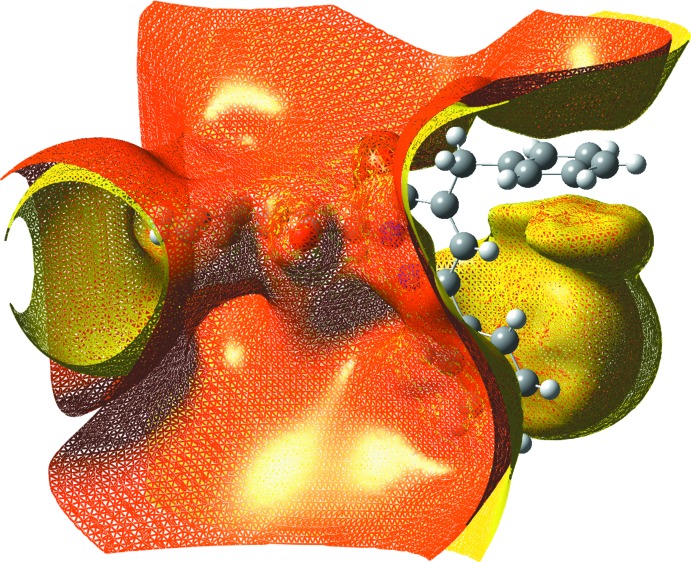
Total electron density mapped over the mol­ecular electrostatic potential surface of the title compound.

The mol­ecular electrostatic potential (MEP) is a technique of mapping electrostatic potential onto the iso-electron density surface. The MEP surface provides information about the reactive sites. The colour scheme is as follows: red for electron rich, partial negative charge; blue for electron-deficient, partial positive charge; light blue for a slightly electron deficient region; yellow for a slightly electron-rich region; green for neutral (Politzer & Murray, 2002 ▸). In addition to these, in the majority of the MEPs, while the maximum positive region, which is the preferred site for nucleophilic attack, is indicated in blue, the maximum negative region, which is the preferred site for electrophilic attack, is indicated in red. The three-dimensional plot of the MEP of the title compound is shown in Fig. 5 ▸. According to the MEP map results, the negative regions of the whole mol­ecule are located on donor oxygen atoms (red regions). The resulting surface simultaneously displays the mol­ecular size and shape and electrostatic potential values. As can be seen from the MEP map contours, regions having negative potential are over the electronegative atoms (*viz*. atoms O1*A* and O2*A* of mol­ecule *A* and O1*B* and O2*B* of mol­ecule *B*). The positive regions are over hydrogen atoms, indicating that these sites are the most likely to be involved in nucleophilic processes.

## Database survey   

A search of the Cambridge Structural Database (CSD, version 5.40, update February 2019; Groom *et al.*, 2016 ▸) gave zero hits for the skeleton of the title compound. A search for pyridazin-3(2*H*)-ones gave 297 hits, while a search for 6-phenyl-pyridazin-3(2*H*)-ones gave 40 hits, including 6-phenyl-pyridazin-3(2*H*)-one itself (CSD refcode CUBBOR; Anderson *et al.*, 2009 ▸). A search for 4-benzyl-6-phenyl-pyridazin-3(2*H*)-ones gave only three hits, for example 4-(4-bromo­benz­yl)-6-phenyl­pyridazin-3(2*H*)-one (VOPMOE; Tsai *et al.*, 2014 ▸). A search for pyridazin-3(2*H*)-ones with an oxoethyl group in position-2 on the pyridazine ring gave eight hits, mostly esters. Four of these structures also have a phenyl substituent in position-6 on the pyridazine ring, as in the title compound. They include, for example 2-(6-oxo-3,4-diphenyl-1,6-di­hydro­pyridazin-1-yl)acetic acid (CIPTOL; Aydın *et al.*, 2007 ▸).

## Synthesis and crystallization   

A mixture of 4-benzyl-6-phenyl­pyridazin-3(2*H*)-one (1 g, 3.8 mmol), K_2_CO_3_ (1.3 g, 9.5 mmol) and 2-chloro-1-(4-fluoro­phen­yl)ethan-1-one (1.58 g, 5 mmol) in acetone (40 ml), was refluxed overnight. The solution was then filtered by suction and the solvent removed under reduced pressure. The residue was purified by recrystallization from ethanol to afford the title compound as colourless prismatic crystals (yield 68%).

## Refinement   

Crystal data, data collection and structure refinement details are summarized in Table 2 ▸. The carbon-bound H atoms were placed in calculated positions (C—H = 0.93–0.97 Å) and included in the refinement in the riding-model approximation, with *U*_iso_(H) = 1.2*U*_eq_(C). The image plate disc in the diffractometer used for the data collection was unfortunately distorted at the outer edges, hence the maximum 2θ value available was limited to 48.8°.

## Supplementary Material

Crystal structure: contains datablock(s) I, Global. DOI: 10.1107/S2056989019008557/su5499sup1.cif

Structure factors: contains datablock(s) I. DOI: 10.1107/S2056989019008557/su5499Isup2.hkl

Supporting information file. DOI: 10.1107/S2056989019008557/su5499Isup3.cml

CCDC reference: 1923216

Additional supporting information:  crystallographic information; 3D view; checkCIF report

## Figures and Tables

**Table 1:** Hydrogen-bond geometry (Å, °) *Cg*1 is the centroid of the N1*A*/N2*A*/C9*A*–C12*A* ring.

*D*—H⋯*A*	*D*—H	H⋯*A*	*D*⋯*A*	*D*—H⋯*A*
C15*A*—H15*A*⋯F1*A* ^i^	0.93	2.49	3.263 (3)	141
C15*B*—H15*B*⋯F1*B* ^ii^	0.93	2.56	3.310 (3)	138
C8*A*—H8*B*⋯O1*A* ^iii^	0.97	2.50	3.466 (3)	179
C8*B*—H8*D*⋯O1*B* ^iv^	0.97	2.49	3.458 (2)	176
C19*A*—H19*A*⋯*Cg*1^iv^	0.97	2.93	3.845 (2)	158

Symmetry codes: (i) 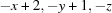; (ii) 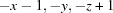; (iii) 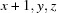; (iv) 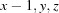.

**Table 2:** Experimental details

Crystal data	
Chemical formula	C_25_H_19_FN_2_O_2_
*M*_r_	398.42
Crystal system, space group	Triclinic, *P* 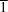
Temperature (K)	296
*a*, *b*, *c* (Å)	5.0575 (3), 10.0973 (7), 38.608 (2)
α, β, γ (°)	86.237 (5), 86.675 (5), 88.354 (5)
*V* (Å^3^)	1963.4 (2)
*Z*	4
Radiation type	Mo *K*α
μ (mm^−1^)	0.09
Crystal size (mm)	0.67 × 0.53 × 0.44

Data collection	
Diffractometer	Stoe IPDS 2
Absorption correction	Integration (*X-RED32*; Stoe & Cie, 2002 ▸)
*T*_min_, *T*_max_	0.953, 0.974
No. of measured, independent and observed [*I* > 2σ(*I*)] reflections	16344, 6363, 4315
*R*_int_	0.031
(sin θ/λ)_max_ (Å^−1^)	0.582

Refinement	
*R*[*F* ^2^ > 2σ(*F* ^2^)], *wR*(*F* ^2^), *S*	0.038, 0.108, 0.99
No. of reflections	6363
No. of parameters	542
H-atom treatment	H-atom parameters constrained
Δρ_max_, Δρ_min_ (e Å^−3^)	0.16, −0.15

Computer programs: *X-AREA* and *X-RED32* (Stoe & Cie, 2002 ▸), *SHELXT2018* (Sheldrick, 2015*a* ▸), *SHELXL2018* (Sheldrick, 2015*b* ▸), *ORTEP-3 for Windows* and *WinGX* (Farrugia, 2012 ▸), *PLATON* (Spek, 2009 ▸) and *Mercury* (Macrae *et al.*, 2008 ▸).

## supplementary crystallographic information

### Crystal data

**Table:**

C_25_H_19_FN_2_O_2_	*Z* = 4
*M**_r_* = 398.42	*F*(000) = 832
Triclinic, *P*1	*D*_x_ = 1.348 Mg m^−^^3^
*a* = 5.0575 (3) Å	Mo *K*α radiation, λ = 0.71073 Å
*b* = 10.0973 (7) Å	Cell parameters from 17316 reflections
*c* = 38.608 (2) Å	θ = 1.1–25.0°
α = 86.237 (5)°	µ = 0.09 mm^−^^1^
β = 86.675 (5)°	*T* = 296 K
γ = 88.354 (5)°	Prism, colourless
*V* = 1963.4 (2) Å^3^	0.67 × 0.53 × 0.44 mm

### Data collection

**Table:**

Stoe IPDS 2 diffractometer	6363 independent reflections
Radiation source: sealed X-ray tube, 12 x 0.4 mm long-fine focus	4315 reflections with *I* > 2σ(*I*)
Plane graphite monochromator	*R*_int_ = 0.031
Detector resolution: 6.67 pixels mm^-1^	θ_max_ = 24.4°, θ_min_ = 1.1°
rotation method scans	*h* = −5→5
Absorption correction: integration (X-RED32; Stoe & Cie, 2002)	*k* = −11→11
*T*_min_ = 0.953, *T*_max_ = 0.974	*l* = −44→44
16344 measured reflections	

### Refinement

**Table:**

Refinement on *F*^2^	Secondary atom site location: difference Fourier map
Least-squares matrix: full	Hydrogen site location: inferred from neighbouring sites
*R*[*F*^2^ > 2σ(*F*^2^)] = 0.038	H-atom parameters constrained
*wR*(*F*^2^) = 0.108	*w* = 1/[σ^2^(*F*_o_^2^) + (0.0633*P*)^2^] where *P* = (*F*_o_^2^ + 2*F*_c_^2^)/3
*S* = 0.99	(Δ/σ)_max_ = 0.001
6363 reflections	Δρ_max_ = 0.16 e Å^−^^3^
542 parameters	Δρ_min_ = −0.14 e Å^−^^3^
0 restraints	Extinction correction: (SHELXL2018; Sheldrick, 2015b), Fc^*^=kFc[1+0.001xFc^2^λ^3^/sin(2θ)]^-1/4^
Primary atom site location: structure-invariant direct methods	Extinction coefficient: 0.0116 (12)

### Special details

**Table:**

Geometry. All esds (except the esd in the dihedral angle between two l.s. planes) are estimated using the full covariance matrix. The cell esds are taken into account individually in the estimation of esds in distances, angles and torsion angles; correlations between esds in cell parameters are only used when they are defined by crystal symmetry. An approximate (isotropic) treatment of cell esds is used for estimating esds involving l.s. planes.

### Fractional atomic coordinates and isotropic or equivalent isotropic displacement parameters (Å^2^)

**Table:**

	*x*	*y*	*z*	*U*_iso_*/*U*_eq_	
F1A	0.6289 (5)	0.09624 (17)	−0.02980 (4)	0.1608 (8)	
O1A	0.1611 (3)	0.54893 (15)	0.06085 (4)	0.0884 (4)	
O2A	0.1102 (3)	0.46400 (14)	0.14261 (3)	0.0843 (4)	
N1A	0.4405 (3)	0.59106 (15)	0.11746 (4)	0.0671 (4)	
N2A	0.5835 (3)	0.70263 (15)	0.11242 (4)	0.0647 (4)	
C1A	0.5570 (7)	0.1845 (3)	−0.00606 (7)	0.1070 (9)	
C2A	0.3566 (7)	0.2716 (3)	−0.01297 (6)	0.1131 (9)	
H2A	0.267897	0.269609	−0.033355	0.136*	
C3A	0.2873 (5)	0.3632 (2)	0.01093 (6)	0.0928 (7)	
H3A	0.147897	0.423052	0.006716	0.111*	
C4A	0.4204 (4)	0.36847 (19)	0.04125 (5)	0.0682 (5)	
C5A	0.6218 (5)	0.2767 (2)	0.04710 (6)	0.0879 (6)	
H5A	0.713023	0.277567	0.067316	0.105*	
C6A	0.6904 (6)	0.1834 (3)	0.02333 (7)	0.1084 (8)	
H6A	0.825601	0.120960	0.027461	0.130*	
C7A	0.3491 (4)	0.47327 (19)	0.06528 (5)	0.0674 (5)	
C8A	0.5252 (4)	0.48597 (19)	0.09515 (5)	0.0716 (5)	
H8A	0.528879	0.402357	0.109027	0.086*	
H8B	0.704330	0.502655	0.085854	0.086*	
C9A	0.2332 (4)	0.56748 (19)	0.14188 (5)	0.0668 (5)	
C10A	0.1890 (4)	0.67082 (19)	0.16591 (4)	0.0643 (5)	
C11A	0.3298 (3)	0.78185 (19)	0.16100 (4)	0.0638 (4)	
H11A	0.296983	0.849831	0.175921	0.077*	
C12A	0.5292 (3)	0.79793 (17)	0.13333 (4)	0.0590 (4)	
C13A	0.6973 (3)	0.91648 (17)	0.12757 (4)	0.0592 (4)	
C14A	0.9022 (4)	0.9212 (2)	0.10193 (5)	0.0701 (5)	
H14A	0.930936	0.850425	0.087826	0.084*	
C15A	1.0634 (4)	1.0296 (2)	0.09714 (5)	0.0785 (5)	
H15A	1.198796	1.031103	0.079764	0.094*	
C16A	1.0263 (4)	1.1351 (2)	0.11770 (5)	0.0771 (5)	
H16A	1.135777	1.207810	0.114378	0.093*	
C17A	0.8272 (4)	1.1320 (2)	0.14305 (5)	0.0800 (6)	
H17A	0.801440	1.202863	0.157184	0.096*	
C18A	0.6631 (4)	1.02435 (19)	0.14795 (5)	0.0737 (5)	
H18A	0.527209	1.024344	0.165248	0.088*	
C19A	−0.0153 (4)	0.6428 (2)	0.19543 (5)	0.0776 (5)	
H19A	−0.189465	0.665208	0.187160	0.093*	
H19B	−0.010103	0.548385	0.201910	0.093*	
C20A	0.0200 (4)	0.7165 (2)	0.22721 (5)	0.0748 (5)	
C21A	−0.1352 (5)	0.8225 (3)	0.23581 (7)	0.1061 (8)	
H21A	−0.268536	0.853174	0.221492	0.127*	
C22A	−0.0946 (9)	0.8864 (3)	0.26656 (11)	0.1434 (13)	
H22A	−0.200439	0.959078	0.272578	0.172*	
C23A	0.1034 (11)	0.8396 (5)	0.28740 (9)	0.1518 (19)	
H23A	0.131088	0.880473	0.307731	0.182*	
C24A	0.2553 (8)	0.7361 (5)	0.27864 (8)	0.1443 (14)	
H24	0.390783	0.705923	0.292665	0.173*	
C25A	0.2138 (5)	0.6744 (3)	0.24941 (6)	0.1052 (8)	
H25A	0.319992	0.600939	0.244112	0.126*	
F1B	−0.1157 (4)	−0.40818 (14)	0.53084 (4)	0.1266 (5)	
O1B	0.3487 (3)	0.07220 (14)	0.43808 (3)	0.0772 (4)	
O2B	0.4021 (3)	0.03214 (13)	0.35481 (3)	0.0802 (4)	
N1B	0.0734 (3)	0.15026 (13)	0.38207 (3)	0.0584 (4)	
N2B	−0.0726 (3)	0.26127 (13)	0.38851 (3)	0.0555 (3)	
C1B	−0.0437 (5)	−0.3100 (2)	0.50691 (5)	0.0843 (6)	
C2B	0.1540 (5)	−0.2291 (2)	0.51344 (5)	0.0873 (6)	
H2B	0.242165	−0.240984	0.533934	0.105*	
C3B	0.2203 (4)	−0.1293 (2)	0.48904 (5)	0.0751 (5)	
H3B	0.356166	−0.073010	0.493036	0.090*	
C4B	0.0889 (3)	−0.11043 (16)	0.45843 (4)	0.0563 (4)	
C5B	−0.1091 (4)	−0.19582 (19)	0.45264 (5)	0.0710 (5)	
H5B	−0.198472	−0.184832	0.432219	0.085*	
C6B	−0.1761 (5)	−0.2978 (2)	0.47698 (6)	0.0842 (6)	
H6B	−0.307935	−0.356518	0.473060	0.101*	
C7B	0.1617 (3)	0.00317 (17)	0.43376 (4)	0.0573 (4)	
C8B	−0.0105 (3)	0.03345 (16)	0.40320 (4)	0.0611 (4)	
H8C	−0.004029	−0.042157	0.388888	0.073*	
H8D	−0.192703	0.046919	0.411803	0.073*	
C9B	0.2882 (3)	0.14027 (18)	0.35847 (4)	0.0601 (4)	
C10B	0.3580 (3)	0.26374 (17)	0.33930 (4)	0.0562 (4)	
C11B	0.2149 (3)	0.37419 (17)	0.34604 (4)	0.0561 (4)	
H11B	0.261247	0.454364	0.334336	0.067*	
C12B	−0.0065 (3)	0.37147 (16)	0.37073 (4)	0.0514 (4)	
C13B	−0.1834 (3)	0.48873 (15)	0.37667 (4)	0.0525 (4)	
C14B	−0.3844 (3)	0.48391 (18)	0.40284 (4)	0.0616 (4)	
H14B	−0.402871	0.407558	0.417523	0.074*	
C15B	−0.5563 (4)	0.59007 (18)	0.40738 (5)	0.0690 (5)	
H15B	−0.689600	0.584914	0.425007	0.083*	
C16B	−0.5320 (4)	0.70395 (19)	0.38596 (5)	0.0683 (5)	
H16B	−0.648412	0.775698	0.389098	0.082*	
C17B	−0.3363 (4)	0.71117 (18)	0.36007 (5)	0.0688 (5)	
H17B	−0.319755	0.787943	0.345510	0.083*	
C18B	−0.1626 (4)	0.60469 (17)	0.35541 (4)	0.0643 (5)	
H18B	−0.029726	0.610886	0.337740	0.077*	
C19B	0.5868 (3)	0.2626 (2)	0.31260 (4)	0.0663 (5)	
H19C	0.697238	0.184128	0.317408	0.080*	
H19D	0.693213	0.339614	0.314856	0.080*	
C20B	0.5043 (3)	0.26336 (16)	0.27574 (4)	0.0559 (4)	
C21B	0.6112 (5)	0.3470 (2)	0.25011 (5)	0.0966 (7)	
H21B	0.740478	0.405093	0.255303	0.116*	
C22B	0.5316 (6)	0.3476 (3)	0.21622 (6)	0.1098 (9)	
H22B	0.606266	0.407224	0.199291	0.132*	
C23B	0.3522 (5)	0.2651 (2)	0.20764 (5)	0.0839 (6)	
H23B	0.302079	0.265730	0.184825	0.101*	
C24B	0.2429 (6)	0.1804 (3)	0.23227 (6)	0.1173 (9)	
H24B	0.115371	0.122164	0.226601	0.141*	
C25B	0.3195 (5)	0.1796 (3)	0.26598 (6)	0.1101 (9)	
H25B	0.242238	0.119871	0.282659	0.132*	

### Atomic displacement parameters (Å^2^)

**Table:**

	*U*^11^	*U*^22^	*U*^33^	*U*^12^	*U*^13^	*U*^23^
F1A	0.256 (2)	0.1202 (12)	0.1074 (11)	−0.0320 (13)	0.0419 (12)	−0.0539 (9)
O1A	0.0780 (9)	0.0997 (11)	0.0865 (9)	0.0114 (9)	−0.0045 (7)	−0.0049 (8)
O2A	0.0967 (10)	0.0754 (9)	0.0813 (9)	−0.0261 (8)	−0.0038 (7)	−0.0001 (7)
N1A	0.0673 (9)	0.0631 (9)	0.0712 (9)	−0.0060 (8)	−0.0003 (8)	−0.0088 (7)
N2A	0.0624 (9)	0.0636 (9)	0.0682 (9)	−0.0079 (8)	−0.0010 (7)	−0.0053 (7)
C1A	0.161 (3)	0.0827 (17)	0.0773 (15)	−0.0335 (18)	0.0291 (16)	−0.0238 (13)
C2A	0.164 (3)	0.104 (2)	0.0745 (15)	−0.040 (2)	−0.0073 (16)	−0.0167 (14)
C3A	0.1055 (17)	0.0920 (15)	0.0825 (14)	−0.0137 (13)	−0.0141 (13)	−0.0050 (12)
C4A	0.0727 (12)	0.0679 (12)	0.0635 (11)	−0.0170 (10)	0.0053 (9)	−0.0026 (9)
C5A	0.1025 (17)	0.0828 (14)	0.0793 (13)	0.0020 (13)	−0.0038 (12)	−0.0167 (11)
C6A	0.130 (2)	0.0906 (17)	0.1048 (19)	0.0070 (15)	0.0092 (16)	−0.0282 (14)
C7A	0.0631 (11)	0.0694 (12)	0.0683 (11)	−0.0107 (10)	0.0063 (9)	0.0019 (9)
C8A	0.0708 (12)	0.0643 (11)	0.0806 (12)	−0.0020 (10)	−0.0027 (10)	−0.0144 (9)
C9A	0.0694 (12)	0.0662 (12)	0.0649 (10)	−0.0094 (10)	−0.0080 (9)	0.0020 (9)
C10A	0.0600 (10)	0.0739 (12)	0.0588 (10)	−0.0085 (10)	−0.0055 (8)	0.0013 (9)
C11A	0.0615 (11)	0.0700 (12)	0.0601 (10)	−0.0042 (9)	−0.0003 (8)	−0.0082 (8)
C12A	0.0568 (10)	0.0637 (11)	0.0567 (9)	0.0001 (9)	−0.0029 (8)	−0.0060 (8)
C13A	0.0563 (10)	0.0643 (11)	0.0574 (9)	−0.0026 (8)	−0.0050 (8)	−0.0041 (8)
C14A	0.0710 (12)	0.0773 (12)	0.0627 (10)	−0.0117 (10)	0.0048 (9)	−0.0126 (9)
C15A	0.0725 (13)	0.0916 (15)	0.0711 (11)	−0.0194 (11)	0.0082 (9)	−0.0056 (11)
C16A	0.0745 (13)	0.0764 (13)	0.0816 (13)	−0.0196 (11)	−0.0055 (11)	−0.0057 (11)
C17A	0.0854 (14)	0.0706 (13)	0.0855 (13)	−0.0117 (11)	0.0015 (11)	−0.0189 (10)
C18A	0.0726 (12)	0.0714 (12)	0.0769 (12)	−0.0078 (10)	0.0097 (10)	−0.0130 (10)
C19A	0.0666 (12)	0.0979 (15)	0.0677 (11)	−0.0175 (11)	0.0021 (9)	−0.0003 (10)
C20A	0.0664 (12)	0.0952 (15)	0.0616 (11)	−0.0213 (11)	0.0083 (9)	0.0020 (10)
C21A	0.0987 (18)	0.1080 (19)	0.1115 (19)	−0.0175 (16)	0.0124 (15)	−0.0148 (16)
C22A	0.171 (3)	0.115 (2)	0.143 (3)	−0.046 (2)	0.055 (3)	−0.041 (2)
C23A	0.202 (5)	0.181 (4)	0.077 (2)	−0.119 (4)	0.030 (2)	−0.023 (2)
C24A	0.154 (3)	0.216 (4)	0.0664 (18)	−0.082 (3)	−0.0151 (17)	0.010 (2)
C25A	0.0970 (17)	0.147 (2)	0.0718 (14)	−0.0226 (16)	−0.0119 (12)	0.0078 (14)
F1B	0.1881 (15)	0.0904 (9)	0.0925 (9)	−0.0023 (10)	0.0239 (9)	0.0314 (7)
O1B	0.0677 (8)	0.0835 (9)	0.0804 (8)	−0.0159 (7)	−0.0044 (7)	0.0024 (7)
O2B	0.0885 (9)	0.0646 (8)	0.0843 (9)	0.0185 (7)	0.0103 (7)	−0.0045 (6)
N1B	0.0605 (8)	0.0512 (8)	0.0615 (8)	0.0053 (7)	0.0018 (7)	0.0024 (6)
N2B	0.0564 (8)	0.0535 (8)	0.0558 (7)	0.0056 (7)	−0.0021 (6)	−0.0003 (6)
C1B	0.1186 (18)	0.0606 (12)	0.0675 (12)	0.0130 (13)	0.0217 (12)	0.0125 (10)
C2B	0.1173 (18)	0.0800 (15)	0.0631 (12)	0.0123 (14)	−0.0109 (12)	0.0060 (11)
C3B	0.0839 (13)	0.0732 (12)	0.0685 (11)	0.0027 (11)	−0.0118 (10)	−0.0017 (10)
C4B	0.0579 (10)	0.0538 (10)	0.0558 (9)	0.0083 (8)	0.0043 (8)	−0.0035 (8)
C5B	0.0781 (13)	0.0672 (12)	0.0667 (11)	−0.0060 (10)	−0.0023 (9)	0.0029 (9)
C6B	0.0966 (15)	0.0675 (12)	0.0861 (14)	−0.0106 (11)	0.0060 (12)	0.0074 (11)
C7B	0.0521 (10)	0.0572 (10)	0.0616 (9)	0.0049 (9)	0.0050 (8)	−0.0062 (8)
C8B	0.0610 (10)	0.0523 (10)	0.0688 (10)	0.0017 (8)	−0.0030 (8)	0.0033 (8)
C9B	0.0604 (11)	0.0611 (11)	0.0586 (9)	0.0077 (9)	−0.0038 (8)	−0.0063 (8)
C10B	0.0531 (9)	0.0658 (11)	0.0500 (8)	0.0006 (8)	−0.0049 (7)	−0.0051 (8)
C11B	0.0561 (10)	0.0578 (10)	0.0537 (9)	−0.0020 (8)	−0.0030 (7)	0.0012 (7)
C12B	0.0527 (9)	0.0532 (9)	0.0484 (8)	−0.0009 (8)	−0.0059 (7)	−0.0011 (7)
C13B	0.0546 (9)	0.0530 (9)	0.0505 (8)	−0.0001 (8)	−0.0078 (7)	−0.0035 (7)
C14B	0.0657 (11)	0.0610 (10)	0.0565 (9)	0.0048 (9)	0.0023 (8)	0.0004 (8)
C15B	0.0708 (12)	0.0685 (12)	0.0660 (10)	0.0085 (10)	0.0074 (9)	−0.0053 (9)
C16B	0.0672 (12)	0.0640 (11)	0.0738 (11)	0.0137 (9)	−0.0071 (9)	−0.0103 (9)
C17B	0.0788 (12)	0.0541 (10)	0.0721 (11)	0.0045 (9)	−0.0055 (10)	0.0042 (8)
C18B	0.0653 (11)	0.0602 (11)	0.0653 (10)	0.0023 (9)	0.0041 (8)	0.0031 (8)
C19B	0.0555 (10)	0.0838 (13)	0.0593 (10)	0.0040 (9)	0.0005 (8)	−0.0065 (9)
C20B	0.0521 (9)	0.0577 (10)	0.0571 (9)	0.0021 (8)	0.0056 (8)	−0.0074 (8)
C21B	0.1225 (19)	0.0981 (16)	0.0714 (13)	−0.0528 (15)	−0.0054 (12)	−0.0004 (11)
C22B	0.166 (3)	0.0959 (17)	0.0681 (13)	−0.0511 (18)	−0.0095 (14)	0.0116 (12)
C23B	0.1031 (16)	0.0903 (15)	0.0592 (11)	0.0007 (13)	−0.0068 (11)	−0.0107 (11)
C24B	0.129 (2)	0.158 (2)	0.0696 (14)	−0.0650 (19)	−0.0042 (13)	−0.0204 (15)
C25B	0.135 (2)	0.134 (2)	0.0635 (13)	−0.0703 (18)	0.0033 (13)	−0.0048 (13)

### Geometric parameters (Å, º)

**Table:**

F1A—C1A	1.348 (3)	F1B—C1B	1.353 (2)
O1A—C7A	1.215 (2)	O1B—C7B	1.217 (2)
O2A—C9A	1.229 (2)	O2B—C9B	1.232 (2)
N1A—N2A	1.353 (2)	N1B—N2B	1.3532 (17)
N1A—C9A	1.384 (2)	N1B—C9B	1.383 (2)
N1A—C8A	1.450 (2)	N1B—C8B	1.449 (2)
N2A—C12A	1.309 (2)	N2B—C12B	1.310 (2)
C1A—C2A	1.349 (4)	C1B—C2B	1.354 (3)
C1A—C6A	1.353 (4)	C1B—C6B	1.366 (3)
C2A—C3A	1.373 (3)	C2B—C3B	1.369 (3)
C2A—H2A	0.9300	C2B—H2B	0.9300
C3A—C4A	1.389 (3)	C3B—C4B	1.389 (2)
C3A—H3A	0.9300	C3B—H3B	0.9300
C4A—C5A	1.375 (3)	C4B—C5B	1.378 (3)
C4A—C7A	1.476 (3)	C4B—C7B	1.483 (2)
C5A—C6A	1.382 (3)	C5B—C6B	1.384 (3)
C5A—H5A	0.9300	C5B—H5B	0.9300
C6A—H6A	0.9300	C6B—H6B	0.9300
C7A—C8A	1.512 (3)	C7B—C8B	1.517 (2)
C8A—H8A	0.9700	C8B—H8C	0.9700
C8A—H8B	0.9700	C8B—H8D	0.9700
C9A—C10A	1.445 (3)	C9B—C10B	1.449 (2)
C10A—C11A	1.342 (2)	C10B—C11B	1.344 (2)
C10A—C19A	1.511 (3)	C10B—C19B	1.505 (2)
C11A—C12A	1.430 (2)	C11B—C12B	1.427 (2)
C11A—H11A	0.9300	C11B—H11B	0.9300
C12A—C13A	1.486 (2)	C12B—C13B	1.485 (2)
C13A—C18A	1.386 (3)	C13B—C18B	1.388 (2)
C13A—C14A	1.391 (2)	C13B—C14B	1.391 (2)
C14A—C15A	1.379 (3)	C14B—C15B	1.374 (2)
C14A—H14A	0.9300	C14B—H14B	0.9300
C15A—C16A	1.372 (3)	C15B—C16B	1.376 (3)
C15A—H15A	0.9300	C15B—H15B	0.9300
C16A—C17A	1.363 (3)	C16B—C17B	1.365 (3)
C16A—H16A	0.9300	C16B—H16B	0.9300
C17A—C18A	1.383 (3)	C17B—C18B	1.383 (2)
C17A—H17A	0.9300	C17B—H17B	0.9300
C18A—H18A	0.9300	C18B—H18B	0.9300
C19A—C20A	1.498 (3)	C19B—C20B	1.505 (2)
C19A—H19A	0.9700	C19B—H19C	0.9700
C19A—H19B	0.9700	C19B—H19D	0.9700
C20A—C21A	1.356 (3)	C20B—C21B	1.356 (3)
C20A—C25A	1.379 (3)	C20B—C25B	1.363 (3)
C21A—C22A	1.416 (5)	C21B—C22B	1.391 (3)
C21A—H21A	0.9300	C21B—H21B	0.9300
C22A—C23A	1.375 (5)	C22B—C23B	1.320 (3)
C22A—H22A	0.9300	C22B—H22B	0.9300
C23A—C24A	1.329 (6)	C23B—C24B	1.342 (3)
C23A—H23A	0.9300	C23B—H23B	0.9300
C24A—C25A	1.354 (4)	C24B—C25B	1.378 (3)
C24A—H24	0.9300	C24B—H24B	0.9300
C25A—H25A	0.9300	C25B—H25B	0.9300
			
N2A—N1A—C9A	126.89 (15)	N2B—N1B—C9B	126.71 (14)
N2A—N1A—C8A	114.62 (14)	N2B—N1B—C8B	113.92 (13)
C9A—N1A—C8A	118.45 (16)	C9B—N1B—C8B	119.37 (13)
C12A—N2A—N1A	117.72 (14)	C12B—N2B—N1B	117.52 (13)
F1A—C1A—C2A	118.3 (3)	F1B—C1B—C2B	119.3 (2)
F1A—C1A—C6A	118.9 (3)	F1B—C1B—C6B	117.6 (2)
C2A—C1A—C6A	122.9 (2)	C2B—C1B—C6B	123.07 (19)
C1A—C2A—C3A	118.2 (2)	C1B—C2B—C3B	118.2 (2)
C1A—C2A—H2A	120.9	C1B—C2B—H2B	120.9
C3A—C2A—H2A	120.9	C3B—C2B—H2B	120.9
C2A—C3A—C4A	121.5 (2)	C2B—C3B—C4B	121.3 (2)
C2A—C3A—H3A	119.2	C2B—C3B—H3B	119.3
C4A—C3A—H3A	119.2	C4B—C3B—H3B	119.3
C5A—C4A—C3A	117.84 (19)	C5B—C4B—C3B	118.68 (17)
C5A—C4A—C7A	122.41 (18)	C5B—C4B—C7B	122.54 (16)
C3A—C4A—C7A	119.72 (19)	C3B—C4B—C7B	118.75 (17)
C4A—C5A—C6A	120.9 (2)	C4B—C5B—C6B	120.48 (19)
C4A—C5A—H5A	119.6	C4B—C5B—H5B	119.8
C6A—C5A—H5A	119.6	C6B—C5B—H5B	119.8
C1A—C6A—C5A	118.7 (3)	C1B—C6B—C5B	118.3 (2)
C1A—C6A—H6A	120.6	C1B—C6B—H6B	120.9
C5A—C6A—H6A	120.6	C5B—C6B—H6B	120.9
O1A—C7A—C4A	121.70 (18)	O1B—C7B—C4B	121.68 (16)
O1A—C7A—C8A	121.01 (17)	O1B—C7B—C8B	120.65 (16)
C4A—C7A—C8A	117.26 (17)	C4B—C7B—C8B	117.65 (16)
N1A—C8A—C7A	113.57 (16)	N1B—C8B—C7B	112.24 (15)
N1A—C8A—H8A	108.9	N1B—C8B—H8C	109.2
C7A—C8A—H8A	108.9	C7B—C8B—H8C	109.2
N1A—C8A—H8B	108.9	N1B—C8B—H8D	109.2
C7A—C8A—H8B	108.9	C7B—C8B—H8D	109.2
H8A—C8A—H8B	107.7	H8C—C8B—H8D	107.9
O2A—C9A—N1A	120.46 (17)	O2B—C9B—N1B	120.15 (16)
O2A—C9A—C10A	125.55 (18)	O2B—C9B—C10B	125.22 (16)
N1A—C9A—C10A	113.95 (17)	N1B—C9B—C10B	114.63 (14)
C11A—C10A—C9A	119.16 (16)	C11B—C10B—C9B	118.57 (15)
C11A—C10A—C19A	124.93 (17)	C11B—C10B—C19B	122.85 (16)
C9A—C10A—C19A	115.91 (17)	C9B—C10B—C19B	118.58 (15)
C10A—C11A—C12A	121.47 (17)	C10B—C11B—C12B	121.60 (15)
C10A—C11A—H11A	119.3	C10B—C11B—H11B	119.2
C12A—C11A—H11A	119.3	C12B—C11B—H11B	119.2
N2A—C12A—C11A	120.52 (17)	N2B—C12B—C11B	120.95 (14)
N2A—C12A—C13A	115.78 (15)	N2B—C12B—C13B	115.81 (14)
C11A—C12A—C13A	123.59 (16)	C11B—C12B—C13B	123.17 (14)
C18A—C13A—C14A	117.33 (17)	C18B—C13B—C14B	117.53 (15)
C18A—C13A—C12A	121.94 (16)	C18B—C13B—C12B	121.44 (14)
C14A—C13A—C12A	120.70 (16)	C14B—C13B—C12B	120.96 (14)
C15A—C14A—C13A	120.89 (18)	C15B—C14B—C13B	121.11 (16)
C15A—C14A—H14A	119.6	C15B—C14B—H14B	119.4
C13A—C14A—H14A	119.6	C13B—C14B—H14B	119.4
C16A—C15A—C14A	120.77 (18)	C14B—C15B—C16B	120.31 (17)
C16A—C15A—H15A	119.6	C14B—C15B—H15B	119.8
C14A—C15A—H15A	119.6	C16B—C15B—H15B	119.8
C17A—C16A—C15A	119.18 (19)	C17B—C16B—C15B	119.71 (17)
C17A—C16A—H16A	120.4	C17B—C16B—H16B	120.1
C15A—C16A—H16A	120.4	C15B—C16B—H16B	120.1
C16A—C17A—C18A	120.60 (19)	C16B—C17B—C18B	120.22 (17)
C16A—C17A—H17A	119.7	C16B—C17B—H17B	119.9
C18A—C17A—H17A	119.7	C18B—C17B—H17B	119.9
C17A—C18A—C13A	121.23 (18)	C17B—C18B—C13B	121.11 (16)
C17A—C18A—H18A	119.4	C17B—C18B—H18B	119.4
C13A—C18A—H18A	119.4	C13B—C18B—H18B	119.4
C20A—C19A—C10A	114.86 (17)	C10B—C19B—C20B	113.81 (14)
C20A—C19A—H19A	108.6	C10B—C19B—H19C	108.8
C10A—C19A—H19A	108.6	C20B—C19B—H19C	108.8
C20A—C19A—H19B	108.6	C10B—C19B—H19D	108.8
C10A—C19A—H19B	108.6	C20B—C19B—H19D	108.8
H19A—C19A—H19B	107.5	H19C—C19B—H19D	107.7
C21A—C20A—C25A	117.7 (2)	C21B—C20B—C25B	115.85 (18)
C21A—C20A—C19A	122.8 (2)	C21B—C20B—C19B	121.93 (17)
C25A—C20A—C19A	119.5 (2)	C25B—C20B—C19B	122.22 (17)
C20A—C21A—C22A	120.1 (3)	C20B—C21B—C22B	121.4 (2)
C20A—C21A—H21A	120.0	C20B—C21B—H21B	119.3
C22A—C21A—H21A	120.0	C22B—C21B—H21B	119.3
C23A—C22A—C21A	119.1 (4)	C23B—C22B—C21B	121.2 (2)
C23A—C22A—H22A	120.4	C23B—C22B—H22B	119.4
C21A—C22A—H22A	120.4	C21B—C22B—H22B	119.4
C24A—C23A—C22A	120.3 (4)	C22B—C23B—C24B	119.1 (2)
C24A—C23A—H23A	119.8	C22B—C23B—H23B	120.4
C22A—C23A—H23A	119.8	C24B—C23B—H23B	120.4
C23A—C24A—C25A	120.3 (4)	C23B—C24B—C25B	120.1 (2)
C23A—C24A—H24	119.8	C23B—C24B—H24B	120.0
C25A—C24A—H24	119.8	C25B—C24B—H24B	120.0
C24A—C25A—C20A	122.4 (3)	C20B—C25B—C24B	122.4 (2)
C24A—C25A—H25A	118.8	C20B—C25B—H25B	118.8
C20A—C25A—H25A	118.8	C24B—C25B—H25B	118.8
			
C9A—N1A—N2A—C12A	2.5 (2)	C9B—N1B—N2B—C12B	0.1 (2)
C8A—N1A—N2A—C12A	−175.39 (15)	C8B—N1B—N2B—C12B	179.13 (14)
F1A—C1A—C2A—C3A	−178.7 (2)	F1B—C1B—C2B—C3B	−179.03 (18)
C6A—C1A—C2A—C3A	0.4 (4)	C6B—C1B—C2B—C3B	1.1 (3)
C1A—C2A—C3A—C4A	0.9 (4)	C1B—C2B—C3B—C4B	0.3 (3)
C2A—C3A—C4A—C5A	−1.4 (3)	C2B—C3B—C4B—C5B	−1.0 (3)
C2A—C3A—C4A—C7A	176.6 (2)	C2B—C3B—C4B—C7B	177.23 (17)
C3A—C4A—C5A—C6A	0.6 (3)	C3B—C4B—C5B—C6B	0.3 (3)
C7A—C4A—C5A—C6A	−177.3 (2)	C7B—C4B—C5B—C6B	−177.79 (16)
F1A—C1A—C6A—C5A	177.9 (2)	F1B—C1B—C6B—C5B	178.42 (17)
C2A—C1A—C6A—C5A	−1.1 (4)	C2B—C1B—C6B—C5B	−1.7 (3)
C4A—C5A—C6A—C1A	0.6 (4)	C4B—C5B—C6B—C1B	0.9 (3)
C5A—C4A—C7A—O1A	−175.73 (19)	C5B—C4B—C7B—O1B	−174.93 (16)
C3A—C4A—C7A—O1A	6.4 (3)	C3B—C4B—C7B—O1B	7.0 (2)
C5A—C4A—C7A—C8A	6.3 (3)	C5B—C4B—C7B—C8B	6.8 (2)
C3A—C4A—C7A—C8A	−171.63 (18)	C3B—C4B—C7B—C8B	−171.34 (15)
N2A—N1A—C8A—C7A	−102.78 (18)	N2B—N1B—C8B—C7B	−99.93 (16)
C9A—N1A—C8A—C7A	79.2 (2)	C9B—N1B—C8B—C7B	79.18 (19)
O1A—C7A—C8A—N1A	2.1 (3)	O1B—C7B—C8B—N1B	−1.2 (2)
C4A—C7A—C8A—N1A	−179.88 (15)	C4B—C7B—C8B—N1B	177.16 (13)
N2A—N1A—C9A—O2A	176.25 (16)	N2B—N1B—C9B—O2B	−179.01 (16)
C8A—N1A—C9A—O2A	−5.9 (3)	C8B—N1B—C9B—O2B	2.0 (2)
N2A—N1A—C9A—C10A	−6.1 (3)	N2B—N1B—C9B—C10B	0.2 (2)
C8A—N1A—C9A—C10A	171.72 (15)	C8B—N1B—C9B—C10B	−178.76 (14)
O2A—C9A—C10A—C11A	−176.77 (17)	O2B—C9B—C10B—C11B	179.82 (17)
N1A—C9A—C10A—C11A	5.7 (2)	N1B—C9B—C10B—C11B	0.6 (2)
O2A—C9A—C10A—C19A	3.9 (3)	O2B—C9B—C10B—C19B	0.1 (3)
N1A—C9A—C10A—C19A	−173.59 (16)	N1B—C9B—C10B—C19B	−179.12 (14)
C9A—C10A—C11A—C12A	−2.4 (3)	C9B—C10B—C11B—C12B	−1.8 (2)
C19A—C10A—C11A—C12A	176.84 (17)	C19B—C10B—C11B—C12B	177.96 (15)
N1A—N2A—C12A—C11A	1.6 (2)	N1B—N2B—C12B—C11B	−1.2 (2)
N1A—N2A—C12A—C13A	177.97 (13)	N1B—N2B—C12B—C13B	175.68 (13)
C10A—C11A—C12A—N2A	−1.5 (3)	C10B—C11B—C12B—N2B	2.2 (2)
C10A—C11A—C12A—C13A	−177.60 (15)	C10B—C11B—C12B—C13B	−174.52 (15)
N2A—C12A—C13A—C18A	−179.53 (16)	N2B—C12B—C13B—C18B	−168.90 (15)
C11A—C12A—C13A—C18A	−3.3 (3)	C11B—C12B—C13B—C18B	8.0 (2)
N2A—C12A—C13A—C14A	−1.6 (2)	N2B—C12B—C13B—C14B	8.0 (2)
C11A—C12A—C13A—C14A	174.69 (17)	C11B—C12B—C13B—C14B	−175.11 (16)
C18A—C13A—C14A—C15A	−0.1 (3)	C18B—C13B—C14B—C15B	0.1 (3)
C12A—C13A—C14A—C15A	−178.21 (17)	C12B—C13B—C14B—C15B	−176.92 (16)
C13A—C14A—C15A—C16A	0.3 (3)	C13B—C14B—C15B—C16B	−0.1 (3)
C14A—C15A—C16A—C17A	−0.1 (3)	C14B—C15B—C16B—C17B	0.1 (3)
C15A—C16A—C17A—C18A	−0.4 (3)	C15B—C16B—C17B—C18B	−0.2 (3)
C16A—C17A—C18A—C13A	0.6 (3)	C16B—C17B—C18B—C13B	0.2 (3)
C14A—C13A—C18A—C17A	−0.3 (3)	C14B—C13B—C18B—C17B	−0.2 (3)
C12A—C13A—C18A—C17A	177.72 (17)	C12B—C13B—C18B—C17B	176.83 (16)
C11A—C10A—C19A—C20A	−24.3 (3)	C11B—C10B—C19B—C20B	−78.2 (2)
C9A—C10A—C19A—C20A	154.93 (18)	C9B—C10B—C19B—C20B	101.59 (18)
C10A—C19A—C20A—C21A	104.7 (2)	C10B—C19B—C20B—C21B	132.6 (2)
C10A—C19A—C20A—C25A	−76.8 (2)	C10B—C19B—C20B—C25B	−47.9 (3)
C25A—C20A—C21A—C22A	0.4 (3)	C25B—C20B—C21B—C22B	1.1 (4)
C19A—C20A—C21A—C22A	178.9 (2)	C19B—C20B—C21B—C22B	−179.4 (2)
C20A—C21A—C22A—C23A	0.0 (4)	C20B—C21B—C22B—C23B	−1.2 (4)
C21A—C22A—C23A—C24A	0.4 (5)	C21B—C22B—C23B—C24B	0.8 (4)
C22A—C23A—C24A—C25A	−1.1 (5)	C22B—C23B—C24B—C25B	−0.4 (4)
C23A—C24A—C25A—C20A	1.5 (5)	C21B—C20B—C25B—C24B	−0.7 (4)
C21A—C20A—C25A—C24A	−1.1 (4)	C19B—C20B—C25B—C24B	179.8 (2)
C19A—C20A—C25A—C24A	−179.7 (2)	C23B—C24B—C25B—C20B	0.4 (5)

### Hydrogen-bond geometry (Å, º)

Cg1 is the centroid of the N1*A*/N2*A*/C9*A*–C12*A* ring.

**Table:**

*D*—H···*A*	*D*—H	H···*A*	*D*···*A*	*D*—H···*A*
C15*A*—H15*A*···F1*A*^i^	0.93	2.49	3.263 (3)	141
C15*B*—H15*B*···F1*B*^ii^	0.93	2.56	3.310 (3)	138
C8*A*—H8*B*···O1*A*^iii^	0.97	2.50	3.466 (3)	179
C8*B*—H8*D*···O1*B*^iv^	0.97	2.49	3.458 (2)	176
C19*A*—H19*A*···*Cg*1^iv^	0.97	2.93	3.845 (2)	158

Symmetry codes: (i) −*x*+2, −*y*+1, −*z*; (ii) −*x*−1, −*y*, −*z*+1; (iii) *x*+1, *y*, *z*; (iv) *x*−1, *y*, *z*.

## References

[bb1] Abiha, G. B., Bahar, L. & Utku, S. (2018). *Rev. Rom. Med. Lab.* **26**, 231–241.

[bb2] Anderson, K. M., Probert, M. R., Whiteley, C. N., Rowland, A. M., Goeta, A. E. & Steed, J. W. (2009). *Cryst. Growth Des.* **9**, 1082–1087.

[bb3] Aydın, A., Doğruer, D. S., Akkurt, M. & Büyükgüngör, O. (2007). *Acta Cryst.* E**63**, o4522.

[bb4] Azaari, H., Chahboune, R., El Azzouzi, M. & Sarakha, M. (2016). *Rapid Commun. Mass Spectrom.* **30**, 1145–1152. 10.1002/rcm.7541 27060843

[bb5] Becke, A. D. (1993). *J. Chem. Phys.* **98**, 5648–5652.

[bb6] Boukharsa, Y., Meddah, B., Tiendrebeogo, R. Y., Ibrahimi, A., Taoufik, J., Cherrah, Y., Benomar, A., Faouzi, M. E. A. & Ansar, M. (2016). *Med. Chem. Res.* **25**, 494–500.

[bb7] Faizi, M. S. H., Gupta, S., Mohan, V. K., Jain, K. V. & Sen, P. (2016). *Sens. Actuators B Chem.* **222**, 15–20.

[bb8] Farrugia, L. J. (2012). *J. Appl. Cryst.* **45**, 849–854.

[bb9] Frisch, M. J., Trucks, G. W., Schlegel, H. B., Scuseria, G. E., Robb, M. A., Cheeseman, J. R., *et al.* (2009). *GAUSSIAN09*. Gaussian Inc., Wallingford, CT, USA.

[bb10] Groom, C. R., Bruno, I. J., Lightfoot, M. P. & Ward, S. C. (2016). *Acta Cryst.* B**72**, 171–179. 10.1107/S2052520616003954 PMC4822653 27048719

[bb11] Ibrahim, T. H., Loksha, Y. M., Elshihawy, H. A., Khodeer, D. M. & Said, M. M. (2017). *Arch. Pharm. Chem. Life Sci.* **350**, e1700093. 10.1002/ardp.201700093 28792072

[bb12] Igarashi, H. & Sakamoto, S. (1994). *J. Pestic. Sci.* **19**, S243–S251.

[bb13] Kamble, V. T., Sawant, A.-S., Sawant, S. S., Pisal, P. M., Gacche, R. N., Kamble, S. S., Shegokar, H. D. & Kamble, V. A. (2017). *J. Basic Appl. Res. Int*, **21**, 10–39.

[bb14] Khokra, S. L., Khan, S. A., Thakur, P., Chowdhary, D., Ahmad, A. & Husain, A. (2016). *J. Chin. Chem. Soc.* **63**, 739–750.

[bb15] Kumar, M., Kumar, A., Faizi, M. S. H., Kumar, S., Singh, M. K., Sahu, S. K., Kishor, S. & John, R. P. (2018). *Sens. Actuators B Chem.* **260**, 888–899.

[bb16] Kumar, S., Hansda, A., Chandra, A., Kumar, A., Kumar, M., Sithambaresan, M., Faizi, M. S. H., Kumar, V. & John, R. P. (2017). *Polyhedron*, **134**, 11–21.

[bb17] Macrae, C. F., Bruno, I. J., Chisholm, J. A., Edgington, P. R., McCabe, P., Pidcock, E., Rodriguez-Monge, L., Taylor, R., van de Streek, J. & Wood, P. A. (2008). *J. Appl. Cryst.* **41**, 466–470.

[bb18] Mukherjee, P., Das, A., Faizi, M. S. H. & Sen, P. (2018). *Chemistry Select*, **3**, 3787–3796.

[bb19] Nauen, R. & Bretschneider, T. (2002). *Pest. Outlook*, **13**, 241–245.

[bb20] Politzer, P. & Murray, J. S. (2002). *Theor. Chim. Acta*, **108**, 134–142.

[bb21] Sheldrick, G. M. (2015*a*). *Acta Cryst.* A**71**, 3–8.

[bb22] Sheldrick, G. M. (2015*b*). *Acta Cryst.* C**71**, 3–8.

[bb23] Spek, A. L. (2009). *Acta Cryst.* D**65**, 148–155. 10.1107/S090744490804362X PMC2631630 19171970

[bb24] Stoe & Cie (2002). *X-AREA*, *X-RED32* and *X-SHAPE*. Stoe & Cie GmbH, Darmstadt, Germany.

[bb25] Tsai, Y.-L., Syu, S.-E., Yang, S.-M., Das, U., Fan, Y.-S., Lee, C.-J. & Lin, W. (2014). *Tetrahedron*, **70**, 5038–5045.

[bb26] Yamada, T., Nobuhara, Y., Shimamura, H., Yoshihara, K., Yamaguchi, A. & Ohki, M. (1981). *Chem. Pharm. Bull.* **29**, 3433–3439. 10.1248/cpb.29.3433 7340942

